# Method for evaluation of human induced pluripotent stem cell quality using image analysis based on the biological morphology of cells

**DOI:** 10.1117/1.JMI.4.4.044003

**Published:** 2017-11-02

**Authors:** Takashi Wakui, Tsuyoshi Matsumoto, Kenta Matsubara, Tomoyuki Kawasaki, Hiroshi Yamaguchi, Hidenori Akutsu

**Affiliations:** a FUJIFILM, Minamiashigara, Japan; b National Research Institute for Child Health and Development, Tokyo, Japan

**Keywords:** induced pluripotent stem cell, cell quality, biological features, visual inspection, machine learning

## Abstract

We propose an image analysis method for quality evaluation of human pluripotent stem cells based on biologically interpretable features. It is important to maintain the undifferentiated state of induced pluripotent stem cells (iPSCs) while culturing the cells during propagation. Cell culture experts visually select good quality cells exhibiting the morphological features characteristic of undifferentiated cells. Experts have empirically determined that these features comprise prominent and abundant nucleoli, less intercellular spacing, and fewer differentiating cellular nuclei. We quantified these features based on experts’ visual inspection of phase contrast images of iPSCs and found that these features are effective for evaluating iPSC quality. We then developed an iPSC quality evaluation method using an image analysis technique. The method allowed accurate classification, equivalent to visual inspection by experts, of three iPSC cell lines.

## 1 Introduction

Since the discovery of human embryonic stem cells (hESCs) by Thomson et al.^1^, researchers have known that hESCs have a specific morphology that differs from that of differentiated cell types. On the other hand, human induced pluripotent stem cells (hiPSCs), established by Takahashi et al.,^2^ have a morphology that is similar to hESCs. Empirically, during the culture process of human pluripotent stem cells (hPSCs) such as hESCs and hiPSCs, a quality check for confirming the undifferentiated state of the cells is performed by assessing the specific morphology of pluripotent stem cells.

The morphological features described in the reports on hPSCs are shown in Table 1. Yu et al.^3^ reported that the specific morphology of ESCs includes a high nucleus:cytoplasm ratio, prominent nucleoli, and formation of compact colonies. Takahashi et al.^2^ also reported features, including formation of round colonies, flat, dense cells, scant cytoplasm, and large nucleoli. Additionally, clear and smooth colony edges and small cells have also been reported.^4^

**Table 1:** Morphological features of hPSCs.

Type	Morphological feature
Colony features	Round
	Compact
	Flat
	Well-defined edge
	Smooth edge
Cell features	High nucleus:cytoplasm ratio
	Prominent nucleoli
	Scant cytoplasm
	Small
	Round

**Fig. 1:**
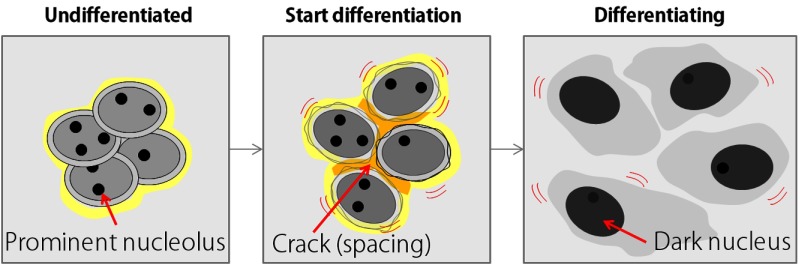
Typical morphological changes in hPSCs during differentiation.

During the general process of culturing hPSCs, culture experts propagate cells while maintaining these morphological features. In the early passage of cells after reprogramming, deviation of cells from the undifferentiated state frequently occurs in the culture due to contamination with cells that were not reprogrammed correctly. Thus, experts must remove these inappropriate cells and keep only the cells that were correctly reprogrammed. When cells deviate from pluripotency toward a differentiated state, they develop a white space that looks like a crack in an intercellular space. Then, the cells gradually develop a dark, flat appearance similar to differentiated cells, and the distance between the cells expands (Fig. 1).^4^ Undifferentiated hPSCs have more relaxed chromatin in their nuclei than do differentiated cells. Because chromatin undergoes a change in structure to heterochromatin,^5^ which causes loss of transparency in nuclei, structures such as nucleoli become unclear and invisible under phase contrast microscopy during the differentiation process.

In general, the pluripotency of hPSCs can be maintained with appropriate quality by checking biological and morphological features through a visual inspection by stem cell culture experts. However, because such an inspection by each individual expert varies, and variation among multiple experts influences the outcome of visual inspection, industrialization of cell production requires a cell evaluation method that is independent of individual expert skills or habits.^6^^–^^8^

Several noninvasive methods based on image analysis have been proposed to replace visual inspection. Many of these methods adopt machine learning technology and classify cells into several quality classes related to visual inspection results with nonmorphological image features, such as the distribution of luminance intensity in cell images.^9^^–^^11^ Maddah et al.^9^ reported that hiPSCs that are classified visually can be adequately distinguished with local binary patterns and an intensity histogram of induced pluripotent stem cells (iPSCs). Tokunaga et al. also showed that cells that were correctly reprogrammed can be distinguished from those that were not using several image features such as the Zernike moment.^10^ In addition, Kato et al.^11^ showed a relationship between image features and gene expression by analyzing the expression of hiPSC colonies classified by using spatial frequency.

As mentioned above, various image analysis methods have been studied, and they all depend on nonmorphological image features. Conventionally, many studies on the culture protocol of hPSCs have shown its adequacy based on the morphology of the cells.^12^^–^^14^ Describing the state of cells using their morphological features enables us to discuss the issues of cell quality and protocol in terms of cell behavior. However, since methods that use image features cannot describe the state of cells, biologists and culture experts cannot easily comprehend problems that arise with target cells, based on the evaluation results of these methods. Hence, development of a cell quality evaluation method employing the morphological features used in the conventional culture process is necessary, without introducing marked changes in the culture process used to establish visual inspection. To achieve this goal, we developed a method for cell quality evaluation that uses the biological morphology of hiPSCs that culture experts especially focus on.

## 2 Methods and Materials

### 2.1 Cell Lines and Culture

In this study, the three hiPSC cell lines MRC5,^15^ Edom,^16^ and 201B7 (Ref. 2) were used. MRC5 and Edom were established in the National Center for Child Health and Development, whereas 201B7 was established by Takahashi et al. Each cell line was cultured in iPSellon medium (Cardio, cat. 007001) supplemented with 10  ng/ml basic fibroblast growth factor (Wako, cat. 068-04544) with mouse embryonic fibroblasts as feeder cells in a six-well microtiter plate. The medium was changed every day. All cell lines were cultured for 5 days so that the confluency of the cells was less than 70%, and therefore, the cells were not too dense.^17^

### 2.2 Cell Imaging Method

**Fig. 2:**
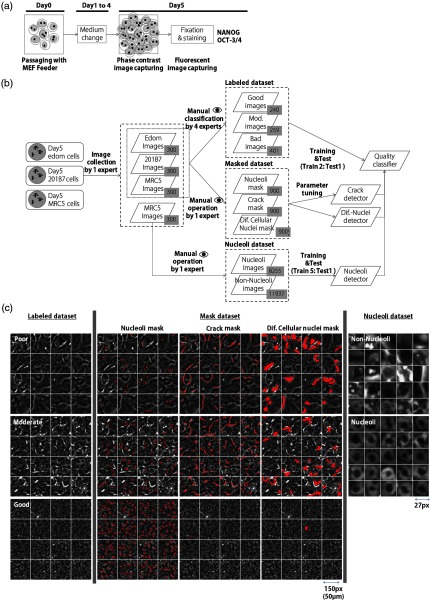
Data acquisition process and datasets. (a) Culture process and image capturing, (b) prepared datasets, and (c) example images of the datasets. In the poor image shown in (c), few differentiating cellular nuclei and crack areas are masked.

The timeline of cell image capturing is shown in Fig. 2(a). Images of the three cell lines were captured 5 days after passage using a phase contrast microscope with a 10× objective lens (Nikon, CFI Plan Fluor DL 10×) and a 3M pixel camera (1920×1440  pixels, Orca 2.8, Hamamatsu Photonics) that can resolve the shapes of the nucleoli in detail. After paraformaldehyde fixation of the same cells, immunofluorescence staining was performed with NANOG (Reprocell, RCAB003P rabbit polyclonal) and OCT-3/4 (Santacruz, SC5279 mouse IgG2b), which are generally used for evaluation of the undifferentiated state. The immunofluorescence images were captured using a confocal microscopy system (Nikon, Eclipse Ti-E) with a 10× objective lens (Nikon, Plan Fluor 10×).

### 2.3 Dataset

The datasets used in this study are shown in Figs. 2(b) and 2(c). We prepared three datasets for the evaluation of cell quality and the development of image analysis algorithms.

First, the original 3M pixel images were divided into small 150×150  pixel images, and 300 small images for the three cell lines were selected by one culture expert. Second, the other four experts evaluated the cell quality of these 900 images and classified them into three classes: poor, moderate (Mod), and good; this is called the labeled dataset. The label that most frequently appeared in the evaluation results of the four experts was defined as the class label for each image. If multiple label candidates existed, the one with lowest quality was selected as the class label.

The second dataset consisted of the training and test images for automatic detection of the biological features from the captured microscopic images. The expert who had selected 900 images inspected all the images manually and marked the three biological features as described below. As a result, 900 masked images were acquired where the locations and regions of the features were marked; this is called the masked dataset.

The third dataset was for the training and test patch images for nucleoli detection. The same expert manually created the nucleoli dataset, in which the size of the patch image was 27×27  pixels, and the nucleolus is located in the center of the patch image. The nucleoli dataset originated from the MRC5 images and differed from the 900 images mentioned above.

## 3 Results

### 3.1 Distinctive Biological Features for Quality Classification of Human Induced Pluripotent Stem Cells

**Fig. 3:**
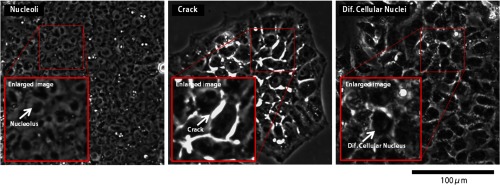
Biological features of hiPSCs.

The aim of this study was the establishment of quality classification of hiPSC images into three classes (poor, Mod, and good) by evaluating the biological features used in the visual inspection. Three features associated with biological structures, namely, the number of nucleoli, the crack area rate, and the differentiating cellular nuclei area rate, were identified by discussion with the experts (Fig. 3). The number of nucleoli is a feature indicating nondifferentiation. Thus, a cell with many nucleoli is considered to be good quality. In contrast, the crack area rate and the differentiating cellular nuclei area rate are indicators of deviation from nondifferentiation. These morphological characteristics have also been reported as features related to the state of iPSCs.^18^ Therefore, the experts considered that these features should be rarely or never detected in hiPSCs with good status. For the purpose of confirming the classification capability of the three features, the distributions of the features for each cell quality class of a respective cell line were investigated.

#### 3.1.1 Variation in scoring of visual inspection results

Variation in scoring among the experts and the cell line-dependent labeled dataset is described in this section.

**Fig. 4:**
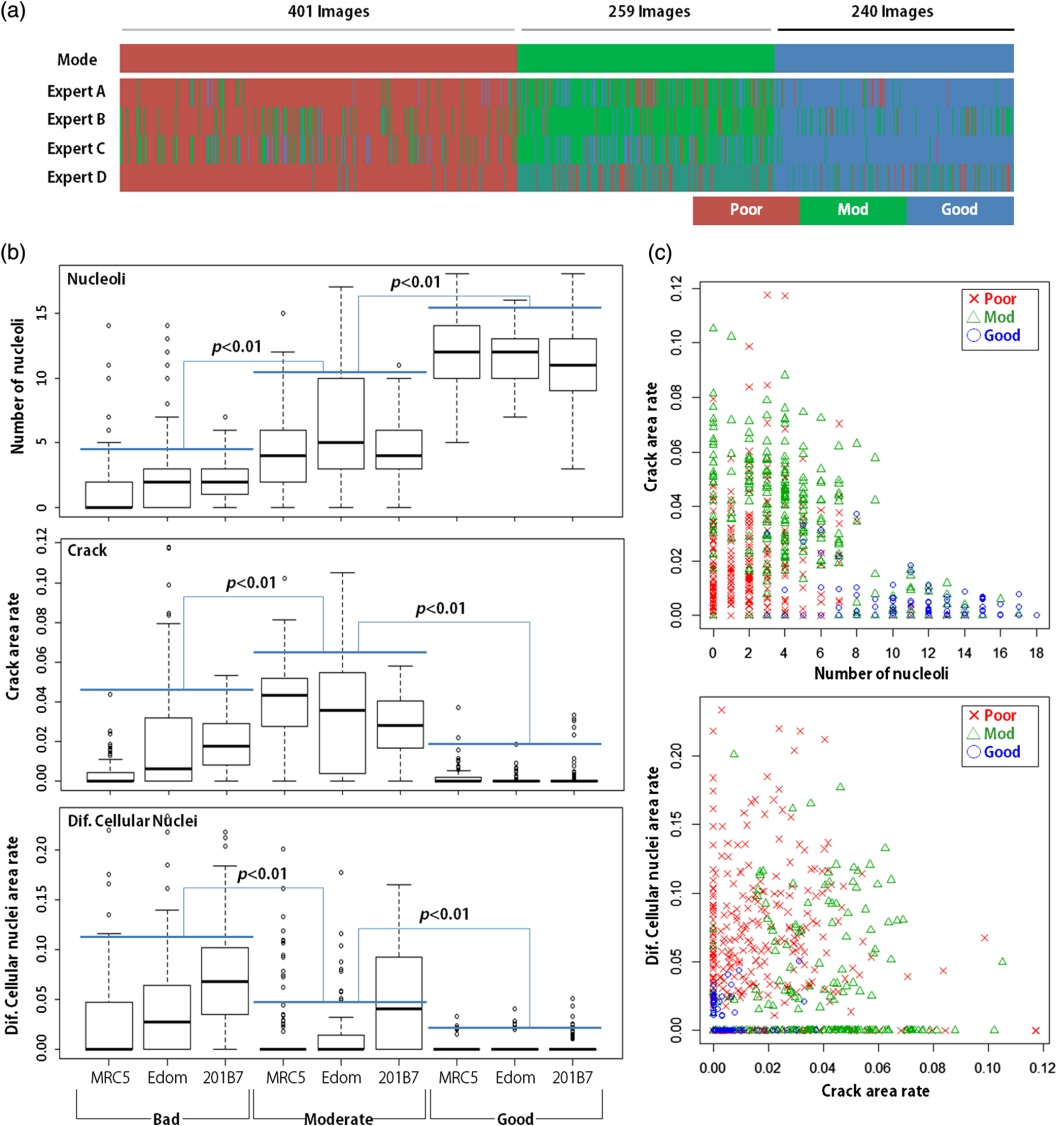
Results of visual inspection of hiPSCs. (a) Interobserver difference for quality classification, (b) the distribution of the features of each cell line and the quality class, and (c) two-dimensional distribution of the features. In (b), each feature was significantly different among the quality classes (p<0.01, with Tukey HSD test).

The interexpert scoring variation is shown in Fig. 4(a) and Table 2. The F-measures were distributed within the range of 0.651 and 0.897, depending on the experts and the cell quality class, the average of which was ∼0.8. The large variation was mainly caused by the widespread distribution of the Mod class. For example, expert A evaluated none as good for all 259 Mod images, whereas expert B evaluated 80 images as good. The Mod class evaluation indicated that the difference in an individual expert’s scoring classification skill was significant for the Mod class compared to the other classes (good and poor).

**Table 2:** Visual inspection performance for cell quality evaluation of the F-measures varied in the range of 0.651 to 0.897 depending on the quality class and the expert. Classification performance for Mod class was worse than the other classes.

	Class	Number	F-measure
Average of the experts	Poor	401	0.874
	Mod	259	0.731
	Good	240	0.810
Expert A	Poor	401	0.897
	Mod	259	0.747
	Good	240	0.730
Expert B	Poor	401	0.834
	Mod	259	0.651
	Good	240	0.799
Expert C	Poor	401	0.872
	Mod	259	0.771
	Good	240	0.850
Expert D	Poor	401	0.893
	Mod	259	0.757
	Good	240	0.861

The cell line dependency of the scoring accuracy is shown in Table 3. The expert-averaged F-measures were distributed widely in the range of 0.630 to 0.826 and 0.664 to 0.892 for the Mod class and the good class, respectively, whereas the poor class was within the range of 0.861 and 0.906. The experts appeared to be able to evaluate the poor class more accurately than the others, regardless of the cell line.

**Table 3:** Cell line dependency of visual inspection performance. The F-measure difference of the poor class was smaller than that of the other classes.

		MRC5		Edom		201B7	
	Class	Number	F-measure	Number	F-measure	Number	F-measure
Average of the experts	Poor	106	0.906	159	0.864	136	0.861
	Mod	117	0.826	84	0.679	58	0.630
	Good	77	0.792	57	0.664	106	0.892
Expert A	Poor	106	0.851	159	0.889	136	0.947
	Mod	117	0.730	84	0.783	58	0.729
	Good	77	0.649	57	0.459	106	0.884
Expert B	Poor	106	0.926	159	0.827	136	0.764
	Mod	117	0.796	84	0.582	58	0.507
	Good	77	0.785	57	0.717	106	0.867
Expert C	Poor	106	0.925	159	0.857	136	0.847
	Mod	117	0.900	84	0.706	58	0.633
	Good	77	0.880	57	0.694	106	0.905
Expert D	Poor	106	0.922	159	0.882	136	0.885
	Mod	117	0.878	84	0.646	58	0.649
	Good	77	0.852	57	0.785	106	0.912

#### 3.1.2 Effectiveness of the biological features for classification

The distributions of the features with respect to each cell quality class [Fig. 4(b)] were investigated in the masked dataset, and the three hypotheses were significantly different. First, the number of nucleoli was significantly increased in accordance with improvement in the cell quality class (p<0.01). Second, the crack area rate was significantly higher in the Mod class (p<0.01). Third, the differentiating cellular nuclei area rate was significantly higher in the poor class (p<0.01). These observations coincided with the result of discussion with the expert, and these features were effective for quality evaluation in the visual inspection.

#### 3.1.3 Classification based on features

Classification based on biological features was investigated. The features of each cell quality class were located in the specific region of the feature space [Fig. 4(c)], in which two of the three appropriate features were selected according to the respective cell quality class. In this feature space, we reclassified each labeled dataset image using a machine learning technique. As shown in Fig. 4(c), the data points distributed on a nonlinear decision hyperplane, which separated each quality class, and the distribution appears to be complicated. Moreover, because the sample size of the labeled dataset is relatively small compared to the size generally used in machine learning applications, over fitting of the training set was a concern. To resolve these issues, we employed a nonlinear support vector machine (SVM) with a radial basis function kernel. This approach has a high generalization ability even for small and complicated datasets, because it employs margin maximization function and a kernel method.^19^^,^^20^

To obtain good classification performance, a grid-search approach was employed to optimize the SVM model for the soft margin constant C and the kernel function parameter gamma (γ). A three-fold cross validation was applied.^21^^–^^23^ As a result of this grid-search optimization, the F-measures of the classification performance were 0.843, 0.683, and 0.831 for the poor, Mod, and good classes, respectively (Table 4), with C=7.94, γ=0.05. Because these numbers are within the range of the numbers generated by visual inspection by the experts, classification with these three features is considered to be sufficiently effective.

**Table 4:** Classification performance using the masked dataset. The performance of the classification using the masked data was equivalent to the average performance of the experts.

	Class	Three cell lines				
		Number	Accuracy	Precision	Recall	F-measure
Automatic classification with mask	Poor	401	0.864	0.872	0.815	0.843
	Mod	259	0.827	0.721	0.649	0.683
	Good	240	0.900	0.757	0.921	0.831

### 3.2 Development of the Image Analysis Method

#### 3.2.1 Image analysis framework

**Fig. 5:**
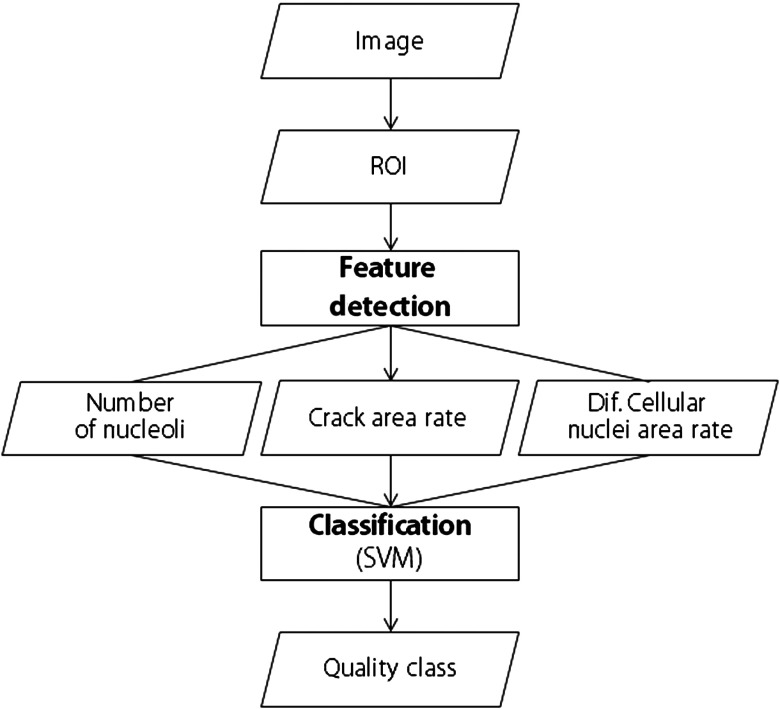
Overview of the proposed method for quality control in the cultivation of hiPSCs.

As explained in the previous section, the three cell quality classes can be distinguished by measuring the three features that experts use in visual inspection. In this section, the image analysis method for iPSC quality evaluation for replacement of the visual inspection process by culture experts is shown, and the flow is described in Fig. 5. The three feature detectors and the cell quality classifier, the inputs of which are the outputs of the detectors, compose the image analysis method, in which the feature detectors and the classifier are applied to each of the regions of interest (150 pixels, 50  μm) for a phase contrast image.

#### 3.2.2 Feature detectors and cell quality classifiers

The details of the feature detectors and the cell quality classifiers are described in this section. For machine learning of the nucleolus detectors, the nucleoli dataset was used as training data. The crack detector and the differentiating cellular nuclei detector were tuned with the masked dataset. The cell quality classifier was developed with the labeled dataset.

The nucleoli detector detects significant, prominent nucleoli in undifferentiated hPSCs. Nucleoli observed in undifferentiated cells are nearly oval-shaped, 3- to 6-μm in diameter, and appear black under phase contrast microscopy. To obtain the robustness of the shape variation of the nucleoli, the nucleolus detector was implemented with the nonlinear SVM classifier,^24^^,^^25^ which classifies a black blob-like region in an image with features such as size and roundness, where the gauss kernel was applied, and then the parameters were optimized with the grid-search technique.

The crack detector detects crack regions that occur between cells during the differentiation process. In many cases, dead cells and stacked regions appear with high luminance intensity in the cell images, appear similar to cracks, and are difficult to distinguish from cracks. On the other hand, because cracks are linear-shaped and different from the shapes of dead cells and stacked regions, a crack region can be detected by extracting a linear-shaped area with high luminance intensity. In this study, the Gabor filter method,^26^ which is frequently used for detecting linear-shaped structures such as blood vessels, was introduced into the crack detector.

In the differentiating cellular nuclei detection process, the nuclear region, which becomes darker after the chromatin structure has changed due to differentiation, is detected. Differentiating cellular nuclei are mostly oval and ∼10-μm in diameter. In this study, the detector detected a differentiating cellular nuclei region by selecting round, black blobs of the indicated sizes after extracting the blobs by the GrabCut method,^27^ which assumes dark and highlighted areas as the source and sink, respectively.

Finally, we trained the cell quality classifier on the nonlinear SVM model with the labeled dataset, in which the SVM kernel was the Gaussian kernel, as shown in Sec. 3.1.3. The kernel parameters C and γ were also optimized by a grid search and we obtained C=0.631, γ=0.126. The labeled dataset was divided into two sets, in which 600 images were for training and the rest were for the cross-validation test.

### 3.3 Image Analysis Performance

As confirmed in Sec. 3.1.3, cell quality classification that is equivalent to visual inspection performance can be achieved if the detector results correlate with the feature detection results of the visual inspection. Therefore, in this section, we investigated the correlation between the feature detectors and the visual inspection results, adding to the performance of the cell quality classifier. Furthermore, the classification performance with respect to each cell line was evaluated and compared with the visual inspection.

#### 3.3.1 Correlation between the detectors and the visual inspection results

**Fig. 6:**
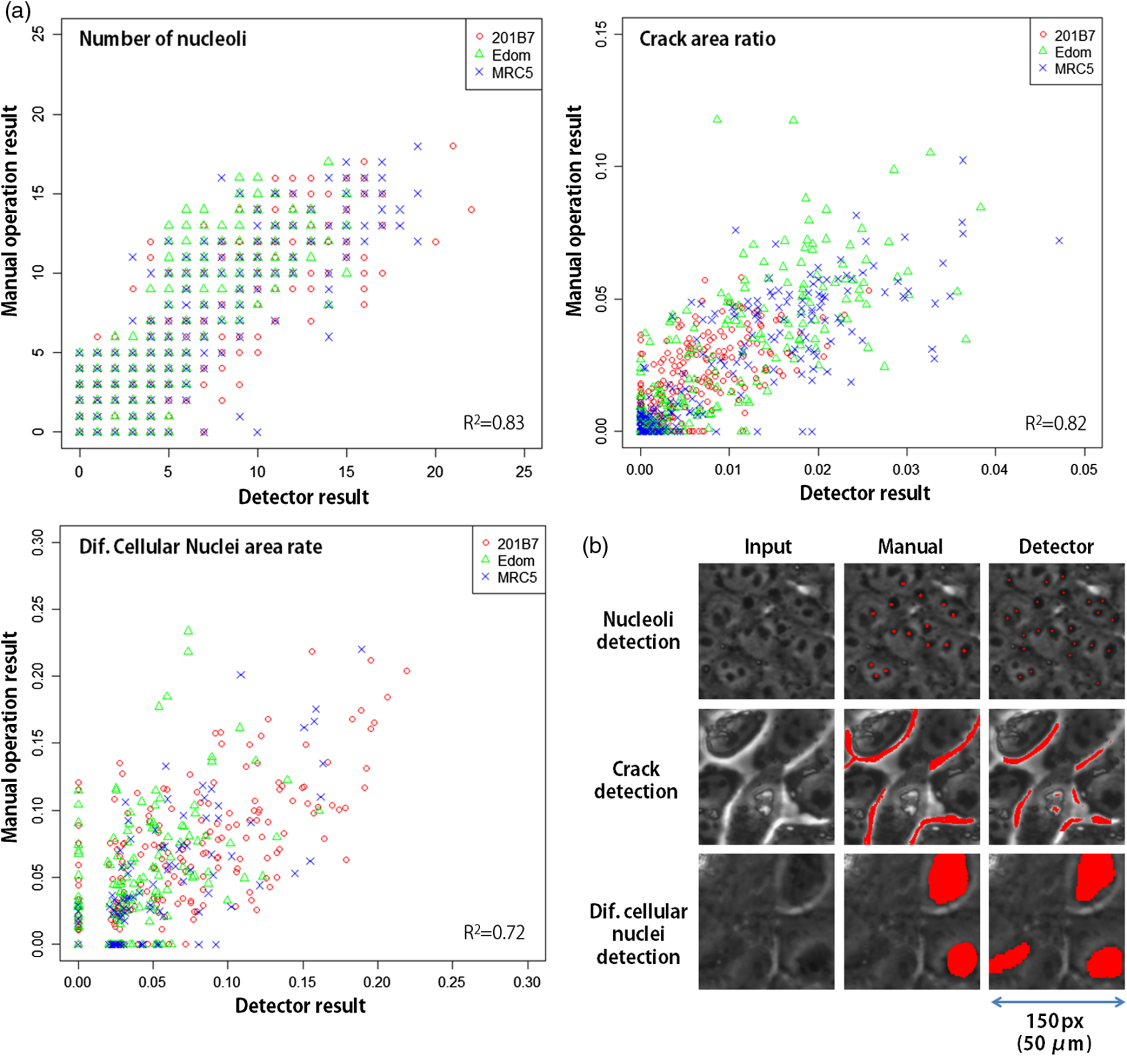
Performance of the developed feature detectors. (a) Correlation between the detectors and the visual inspection results with respect to the cell lines and (b) examples of the detection results. In (b), the red area indicates the area detected by the expert and the detectors. The differences in the manual and detector images are due to errors by the detectors.

The correlation between the outputs of the detectors and the visual inspection results of the experts was investigated [Fig. 6(a)]. The correlation between the detectors was $R^{2}=0.83$, 0.82, and 0.72 for the nucleoli detector, the cracks, and the differentiating cellular nuclei, respectively. The performance of the nucleoli detector on the nucleoli dataset was F-measure=0.985 and 0.982 for training and testing, respectively.

#### 3.3.2 Performance of the cell quality classifier

The performance of the cell quality classifier was evaluated with 300 test images of the labeled dataset (Table 5). The F-measures calculated with data from the three cell lines were 0.820, 0.686, and 0.812 for poor, Mod, and good classes, respectively. These measures performed close to the result of expert B and the result when using the manually masked data mentioned in Sec. 3.1.3.

**Table 5:** Performance of the cell quality classifier. The performance of the cell quality classifier was close to that of the experts. In addition, the performance for Edom was lower than that for the other cell lines because of the cell line dependency.

	Class	Three cell lines				
		Number	Accuracy	Precision	Recall	F-measure
Training (three cell lines)	Poor	266	0.810	0.768	0.820	0.793
	Mod	172	0.810	0.693	0.605	0.646
	Good	162	0.870	0.753	0.772	0.762
Test (three cell lines)	Poor	135	0.840	0.832	0.807	0.820
	Mod	87	0.823	0.707	0.667	0.686
	Good	78	0.897	0.770	0.859	0.812
Test (MRC5)	Poor	36	0.860	0.775	0.861	0.816
	Mod	39	0.890	0.938	0.769	0.845
	Good	25	0.890	0.750	0.840	0.792
Test (Edom)	Poor	53	0.800	0.902	0.698	0.787
	Mod	28	0.750	0.548	0.607	0.576
	Good	19	0.870	0.607	0.895	0.723
Test (201B7)	Poor	46	0.860	0.820	0.891	0.854
	Mod	20	0.830	0.579	0.550	0.564
	Good	34	0.930	0.935	0.853	0.892

#### 3.3.3 Cell line dependencies of the cell quality classifier

The difference in discrimination performance for each cell line was investigated and compared with the visual inspection results. Calculating the F-measures after reclassifying Sec. 3.3.2 result with respect to each cell line, the F-measures were 0.816, 0.845, and 0.792 for the three quality classes of MRC5, 0.787, 0.576, and 0.723 for Edom, and 0.854, 0.564, and 0.892 for 201B7, respectively. The performance of the cell quality classifier was close to the range of the results of the four experts. A difference in performance was noted among the cell lines, similar to the visual inspection results.

## 4 Discussion

As described above, our method achieved accuracy for cell quality classification that was equivalent to visual inspection with respect to the three hiPSC cell lines, and thus replacing the conventional visual inspection process with the image analysis method was possible.

One issue to be addressed before replacement is the cell line dependency of the performance of the cell quality classification. The difference in the feature distribution among the cell lines that caused these dependencies is shown in Fig. 4(b). For robust and highly accurate evaluation that is independent of cell lines, the evaluation method may require feature normalization for each cell line to correct the difference between cell lines. This is because the order of the quality classes was observed in one cell line, but their quality value distributions changed from cell line to cell line.

**Fig. 7:**
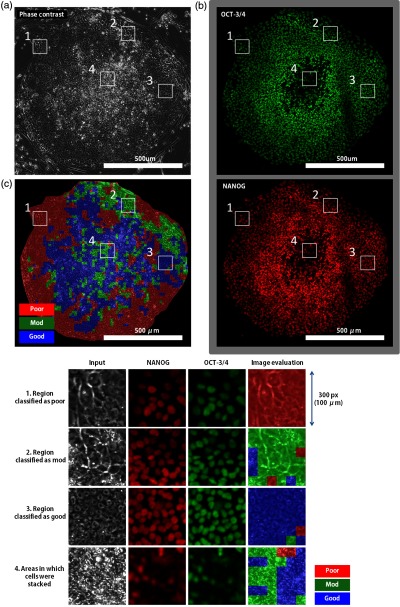
Comparison between the image evaluation result and the immunostaining result. (a) Input phase contrast image, (b) images following immunostaining with OCT-3/4 and NANOG, which are markers of nondifferentiation, and (c) the image evaluation result, and a closed-up comparison of each result. The image evaluation result and the immunostaining result correspond to poor and good regions, respectively, except for the area in which cells were stacked (center of the images).

Another issue regarding the actual culture process is that immunostaining with nondifferentiation markers is generally used for the final inspection, whereas visual inspection is conducted daily for quality check. Similarly, combining our method with immunostaining inspection would be preferred, because the image evaluation method, whether using the image analysis method or visual inspection, does not always correspond to the immunostaining result regarding the nondifferentiation state. Examples of images analyzed with both methods are shown in Fig. 7. In Figs. 7(a) and 7(b), the undifferentiated cells appeared to be distributed in a donut-like shape, because the marker emission was weak in the colony periphery and center where the cells were stacked. The result shown in Fig. 7(c) was acquired by evaluating this image by scanning a region of interest every 50 pixels. The colony periphery where the markers emitted weaker signals tended to be evaluated as poor, whereas the donut-like area where the markers emitted strong signals tended to be evaluated as good. The image evaluation result appeared to correspond to the result of the undifferentiated immunostaining in good and poor images.

However, the colony center and the upper right areas, which were mostly evaluated as Mod, emitted strong and weak signals, respectively. The Mod areas did not correspond to marker emission. Thus, the image evaluation method did not necessarily correspond to the immunostaining inspection result for identifying undifferentiated cells, and replacing inspection after immunostaining with the image evaluation method appears difficult. Therefore, use of the image evaluation method together with the immunostaining inspection method will be necessary to obtain results similar to the conventional method. Other biological features, such as the features described in Table 1, need to be added to represent the immunostaining result. In addition, it is also important to identify the biological mechanisms responsible for imparting the visible morphological characteristics to the cells.

## 5 Conclusion

In this study, we developed a method for the evaluation of cell quality that focused on the biological features of hiPSCs. According to discussion with culture experts, the three biological features that the experts used in the visual inspection process were specific, and then the relationship between the features and the results of cell quality evaluation was investigated. We also developed feature detectors and a cell quality classifier and found that our evaluation method accurately evaluated cells with a result that was equivalent to visual inspection (F-measure ∼0.80). To achieve complete image analysis evaluation, further research on new biological features is necessary to analyze various markers of nondifferentiation and differentiation for use in inspection following immunostaining.
